# KIR genes and adult T-cell leukemia/lymphoma in patients infected with HTLV-1

**DOI:** 10.1186/1742-4690-12-S1-P16

**Published:** 2015-08-28

**Authors:** Omar Melo, Eduardo Gotuzzo, Giovanni Lopez, Jason Rosado, Michael Talledo

**Affiliations:** 1 Laboratorio de Epidemiología Molecular, Universidad PeruanaCayetano Heredia, Lima, Perú; 2 Instituto de Medicina Tropical Alexander von Humboldt, Universidad Peruana Cayetano Heredia, Lima, Perú; 3 Facultad de Medicina, Universidad PeruanaCayetano Heredia, Lima, Perú; 4 Hospital Nacional Cayetano Heredia, Lima, Perú

## 

Adult T-cell leukemia/lymphoma (ATLL) is an aggressive neoplastic disease whose etiologic agent is HTLV-1. ATLL patients have a pronounced immunosuppression of innate and adaptive immune system, compared to asymptomatic carriers. Killer-cell immunoglobulin-like receptors (KIR) play an important role in the signaling of natural killer cells and also are involved in the activity of CD8+ T cells, therefore these have a great repercussion in the innate and adaptive immune response. According to their intracytoplasmic motif they have an activating or inhibiting function on the cells that express these receptor. KIRs are expressed by a family of 15 genes highly polymorphic in the number of both alleles and genes present in the genome, with respect to the latter have been described haplotypes A and B. To assess the association of KIR genes with the presence of ATLL, 14 genes KIR were genotyped using PCR-SSP. For this purpose, genomic DNA was isolated from PBMCs from 147 unrelated individuals with HTLV-1 (20 ATLL and 127 asymptomatic carriers). Odds ratios (OR) were obtained by cross products and their statistical significance using Fisher's exact Test, with 95% confidence level. To determine whether this association is independent of the proviral load (PVL), known risk factor for ATLL, logistic regression was performed with and without PVL. Association of individual KIR genes with the presence of ATLL wasn't found, but an association between AA dyplotype and the presence of ATLL (odds ratio = 3.48, confidence interval 95% = 1.15-11.69, P = 0.02) was found. A loss of statistical significance was observed when the PVL is incorporated into the logistic model (odds ratio = 2.15, confidence interval 95% = 0.65-7.12, P = 0.21), which indicates that the association between genotype AA and ATLL is not independent of proviral load. We conclude that there is not statistically significant evidence to associate KIR genes with ATLL.

